# Visuomotor Adaptation: How Forgetting Keeps Us Conservative

**DOI:** 10.1371/journal.pone.0117901

**Published:** 2015-02-27

**Authors:** Katinka van der Kooij, Eli Brenner, Robert J. van Beers, Jeroen B. J. Smeets

**Affiliations:** MOVE Research Institute Amsterdam, Faculty of Human Movement Sciences, VU University Amsterdam, Amsterdam, The Netherlands; University of California, Merced, UNITED STATES

## Abstract

Even when provided with feedback after every movement, adaptation levels off before biases are completely removed. Incomplete adaptation has recently been attributed to forgetting: the adaptation is already partially forgotten by the time the next movement is made. Here we test whether this idea is correct. If so, the final level of adaptation is determined by a balance between learning and forgetting. Because we learn from perceived errors, scaling these errors by a magnification factor has the same effect as subjects increasing the amount by which they learn from each error. In contrast, there is no reason to expect scaling the errors to affect forgetting. The magnification factor should therefore influence the balance between learning and forgetting, and thereby the final level of adaptation. We found that adaptation was indeed more complete for larger magnification factors. This supports the idea that incomplete adaptation is caused by part of what has been learnt quickly being forgotten.

## Introduction

If our tennis ball consistently hits the court to the right of the sideline, we realize that our movements are biased. It is well known that in such cases we readily adjust our movement planning so that performance errors are reduced, a phenomenon called adaptation (see [1–3] for reviews). A fascinating phenomenon has repeatedly been observed, but has not received much attention: adaptation levels off before biases are completely removed [4–14]. This is especially clear when subjects can only see their errors after their movement has ended (as in the tennis example), but even when subjects receive continuous visual feedback, they only partially adjust their movement plan and then correct for the remaining bias on the fly (e.g. [9]). It is important to understand why adaptation is incomplete, because incomplete adaptation implies that when sudden changes in our sensorimotor system (for instance due to injury) induce movement biases, we can never fully recover. If we understand why adaptation is normally incomplete, we may be able to develop rehabilitation methods that increase the final level of adaptation. In this paper we address the question why adaptation is incomplete and demonstrate how the point at which adaptation levels off can be shifted.

Adaptation is generally studied by measuring how performance errors change after a perturbation is introduced using prism glasses, perturbed virtual visual feedback or mechanical perturbations (see [1–3] for reviews). The results show that subjects deal with biases by correcting for a fraction of the observed error on each trial [1,4,8,15–17]. Doing so should result in biases eventually being removed completely, but this does not always happen (e.g. [4–14]). Moreover, when there is no feedback to learn from, because the visual feedback is removed (e.g. [14,18,19]) or manipulated to indicate that there are no errors regardless of performance (‘error clamp’; [5,13,20]), errors gradually drift back towards a baseline state, a phenomenon that cannot be explained by learning alone.

To accommodate drift towards a baseline state, recent models of adaptation combine error-based learning with a constant return to baseline, which we refer to as ‘forgetting’ [5,13,19]. In these models, subjects adjust for a fraction (learning fraction) of the previous error on each trial and retain only a fraction (retention fraction) of what had been learnt from all previous trials. In contrast to models involving only learning, these models can account for the finding that adaptation is sometimes incomplete, because the forgetting pulls errors back towards the baseline state [21,22]. For a single learning fraction and retention fraction, one might expect errors to return to baseline exponentially when there is no feedback to learn from. However, errors appear to level off at a percentage of what has been learnt from the feedback [13,14]. This can be accommodated by adaptation occurring at multiple timescales: one process learns and forgets rapidly while another learns and forgets slowly. The forgetting by the fast process dominates the errors’ initial fast return towards baseline upon removal of feedback. What the slow process learnt determines the point at which errors appear to level off [13].

Models that incorporate a ‘forgetting’ parameter described data from adaptation studies so adequately that the idea that adaptation is limited by forgetting was readily accepted. Yet the idea that adaptation is limited by forgetting has deep implications for how we interpret human performance. It implies that human performance is either inherently biased [23,24] or that unbiased performance must be achieved through other mechanisms [24]. Understanding the factors that limit adaptation also has applications in the development of rehabilitation programs that aim to reduce movement biases, for instance following injury.

Here we test the idea that the level of adaptation is determined by a balance between learning and forgetting. If the level of adaptation is determined by such a balance, we should be able to change the level of adaptation by perturbing this balance. We perturb this balance by scaling the errors. Magnifying the errors can be expected to enhance learning, whereas forgetting is assumed to be independent of the observed errors [13]. Magnifying the errors therefore causes the learning to have a proportionally greater influence on the adaptation, and thereby improves the asymptotic level of adaptation.

## Methods

### Ethics statement

The experiment was conducted in accordance with the Declaration of Helsinki and was part of an ongoing research program for which consent procedures were approved by the ethics committee of the Faculty of Human Movement Sciences. All subjects gave written informed consent by signing an informed consent document. All data were encoded and analyzed anonymously.

### Subjects

Thirteen subjects (two male, eleven female) participated in the experiment. All subjects were PhD students or postdoctoral researchers at the Faculty of Human Movement Sciences at the VU University Amsterdam, who participated voluntarily and were unaware of the purpose of the experiment.

### Set-up

**Fig 1:**
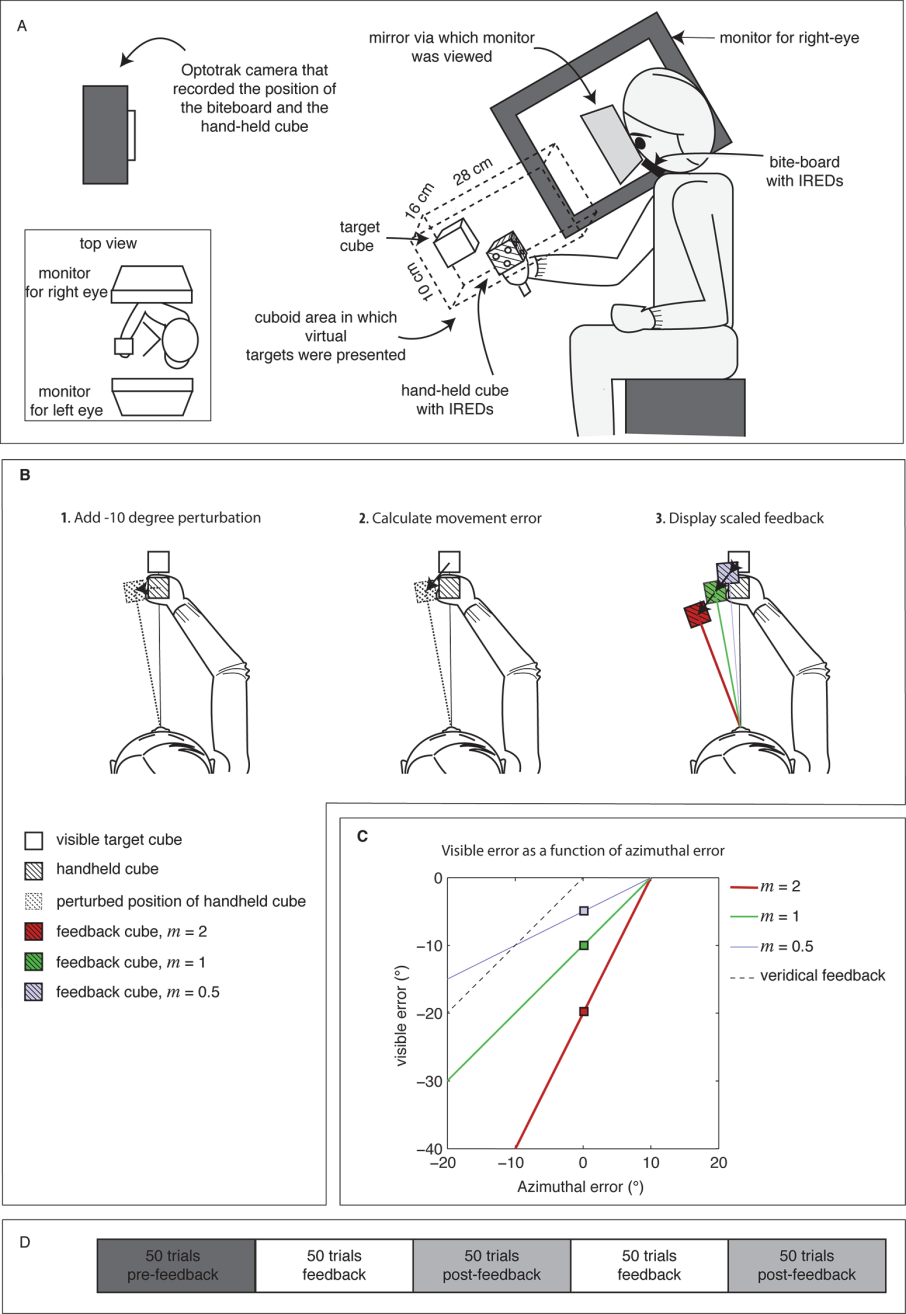
Methods. **(A)** Experimental set-up. **(B)** Illustration of the three steps in which movement errors were perturbed and scaled. The examples are made for a 0° azimuthal error combined with a 6 cm undershoot in depth. Colors correspond to the magnification factor *m*. **(C)** Dependency of the visible errors on the azimuthal errors for the three magnification factors. The colored squares correspond to those in panel B.3. **(D)** Sequence of block types (pre-feedback, feedback, and post-feedback) within a session.

The set-up was the same as the one used in an earlier study [14]. Subjects were seated in a dark room, where they viewed a separate CRT display (48 x 31 cm; viewing distance about 40 cm; resolution 1096 x 686 pixels, 160 Hz) with each eye via mirrors (Fig. 1.A). Infra-red emitting diodes (IREDs) were mounted on a cube with 5-cm edges that subjects held in their right hand and that allowed us to track the movements of the subject’s hand at 100 Hz with an Optotrak 3020 motion analysis system (NDI, Waterloo, ON). The cube was attached to a grip that subjects held, so we could render a floating cube without subjects expecting to see their fingers occluding the surface of the cube.

To be able to render an adequate image of the scene without having to restrain the subject’s head, IREDs were also mounted on a bite-board that subjects held in their mouth and that was not connected to the set-up. For each subject, we determined the eyes’ locations relative to the bite-board as described in detail elsewhere [25]. Knowing the eyes’ locations relative to the tracked bite-board allowed us to render an appropriate new image of the 3D scene for each eye with a latency of about 25 ms between subjects’ movements and the corresponding update of the display.

### Task

Subjects were asked to bring the handheld cube to the position of red virtual target cubes of the same dimensions. The subjects were told that they would sometimes see the handheld cube in blue after a movement had ended, and at other times they would receive no visual feedback about their hand’s position. Target cubes appeared in pseudo-random directions with an inter-target distance of 20 cm. The positions were selected such that they remained within a 16 by 10 by 28 cm cuboid area oriented slightly downward with the longest edge along the line of sight (Fig. 1.A).

A trial started with the appearance of a red target cube, at which time the subject could initiate his movement. When the speed of the handheld cube had been below 2 cm/s for 300 ms we registered the position at that moment to be the movement endpoint. In trials without visual feedback, the next target appeared as soon as the movement had ended. In trials with visual feedback, a static blue feedback cube was shown for 500 ms at the position that depended on where the movement had ended. After that, the next target appeared and subjects moved on (from their latest endpoint) to this new target.

### Visual feedback

To study how subjects use visual feedback about their errors to improve later movements, the visual feedback about the handheld cube was manipulated in three steps (Fig. 1.B).

First, to introduce a performance bias to which subjects had to adapt, we introduced a 10-degree leftward rotational perturbation to the feedback about the hand’s position (Fig. 1.B.1). Due to this perturbation, subjects got visual feedback that the target and hand-held cube were aligned when they actually stopped 10° to the right of the visible target cube (azimuthal angle with respect to the cyclopean eye; positive is to the right). Subjects were not informed about this perturbation. In order to scale the error, we calculated the movement error, which we defined as the 3D vector between the perturbed position of the hand-held cube and the target position (Fig. 1.B.2). Finally, to study how error magnification affects adaptation, we scaled this movement error by a magnification factor *m* (Fig. 1.B.3; Fig. 1.C). In a reduced error condition, *m* was 0.5; in the true-size condition, *m* was 1; and in the magnified condition, *m* was 2. Although the perturbation was only in the azimuthal direction, we scaled all three components of the movement error to keep the shape of the random error distribution constant across conditions. Together this resulted in visible errors that depended with a constant offset but different gain on the azimuthal errors (Fig. 1.C). Thus note that errors were scaled but the amount that subjects had to adapt did not change.

### Procedure

There were three types of blocks of trials. Each block consisted of 50 trials (Fig. 1.D). From a *pre-feedback block*, in which no feedback cubes were shown, we determined baseline biases and standard deviations in movement errors. From subsequent *feedback blocks*, we measured adaptation to the perturbed feedback. From the following *post-feedback* blocks, in which subjects again moved without any visual feedback, we measured the forgetting. The three magnification conditions (m = 2, m = 1 and m = 0.5) were presented in different sessions that lasted about 12 minutes. Each session started with a pre-feedback block, followed by two replications of a feedback and a post-feedback block.

We used a within-subjects design in which each subject performed all three magnification conditions, which allowed us to model the effect of the magnification on the adaptation for each subject. Having subjects perform all sessions also means that there may be some transfer of learning between sessions, so that subjects relearn the perturbation faster than they learnt it the first time (‘savings’, [27]) or retain part of what they have learnt [14]. To randomize a possible effect of savings between sessions, sessions were performed in an approximately counter-balanced order. Moreover, sessions were performed with a minimum 24-hour inter-session interval.

### Data analysis & model

Although movement errors were scaled in all three directions, we chose to analyze errors only in the azimuthal directions, which is the direction in which the perturbation was introduced. We chose to do so because subjects may have large natural biases that they correct for differently than for biases that have been imposed by a perturbation [14]. When we report azimuthal errors (*θ*), these are the true differences between the azimuthal directions of the hand-held and visible target cubes at the movements’ endpoints, with directions expressed relative to the cyclopean eye. So, if subjects successfully corrected for the -10° rotation of the target, we would see azimuthal errors of 10° (we define rightward rotation to be positive). If subjects did not adapt to the -10° visual rotation at all, azimuthal errors would only reflect naturally occurring biases [14]. We refer to the errors between the visual feedback and target cubes as visible errors.

We used as our first measure of the level of adaptation the mean azimuthal error in the last ten trials of a feedback block. To determine whether the level of adaptation depended on the magnification and on repetition of the feedback block, the individual subjects’ levels of adaptation were entered in a 3 x 2 repeated measures analysis of variance with the factors Magnification (2, 1, 0.5) and Repetition (1,2).

To study how learning and forgetting contributed to the adaptation in more depth, we used the model of adaptation proposed by Smith et al. (2006). In this model, adjustments to the movement planning on trial *i* are determined by two parallel processes: one that learns rapidly and retains little and one that learns slowly but has good retention. Each process adjusts a state (*S*_1_, *S*_2_) on the basis of the azimuthal visible errors (*e*_i_), which are the differences in the azimuthal directions of the target and feedback cube relative to the cyclopean eye. Each process has its own learning fraction (*B*_1_, *B*_2_) and retains the state with its own retention fraction (*A*_1_, *A*_2_). Azimuthal errors (*θ*) are determined by the sum of the two states and are described by:

$$S1_{i+1}=A_{1}\cdot S1_{i}+B_{1}\cdot e_{i}$$ [1.a]

$$S2_{i+1}=A_{2}\cdot S2_{i}+B_{2}\cdot e_{i}$$ [1.b]

$$\theta_{i}=S1_{i}+S2_{i}$$ [1.c]

Since subjects did not receive visual feedback in the pre-feedback and post-feedback blocks, *e*_i_ was set to zero for these blocks. Thus, the errors during such blocks only depended on the initial states and the retention fractions. In feedback blocks, we manipulated the visual feedback (*e*_i_) about the azimuthal errors (*θ*_i_) by adding a 10° perturbation of the target and scaling the resulting error by a magnification factor (*m*). To include these influences of the perturbation and magnification factor on the errors, we set *e*_i_ in blocks with feedback to:

$$e_{i}=m\cdot (\theta_{i}-10)$$ [2]

This way, without forgetting, the asymptotic level of adaptation would be complete for all three magnification conditions. With forgetting, the asymptotic level of adaptation would be highest for the largest magnification factor and lowest for the smallest magnification factor, but always incomplete.

When subjects move without visual feedback about their hand’s position, they have small pre-existing biases that keep reappearing after blocks of visual feedback have been presented [14,19]. We estimated these biases from the mean azimuthal error in the pre-feedback blocks, disregarding the first 5 trials, which were considered practice trials. The definition of *θ*_*i*_ in equation 1.C is therefore rewritten to include these biases (*b*) as a constant element of the azimuthal error:

$$\theta_{i}=S1_{i}+S2_{i}+b$$ [3]

This way, complete adaptation would result in azimuthal errors of 10° and removing the visual feedback would cause the azimuthal errors to drift towards the pre-existing bias (*θ = b*).

The 4-parameter model was simultaneously fit to the data of the full feedback and post-feedback block sequence. This was done for individual subjects, considering all feedback and post-feedback blocks, using MatLab’s nlinfit function, a nonlinear regression that estimates the parameters using iterative least squares estimation. This was first done for each magnification condition separately, and later for all magnification conditions together. There were no constraints on the parameters. Although the two processes are mathematically equivalent, we will consider the one with the highest value of *B* to be the first process and refer to it as the fast process. We refer to the other process as the slow process.

## Results

**Fig 2:**
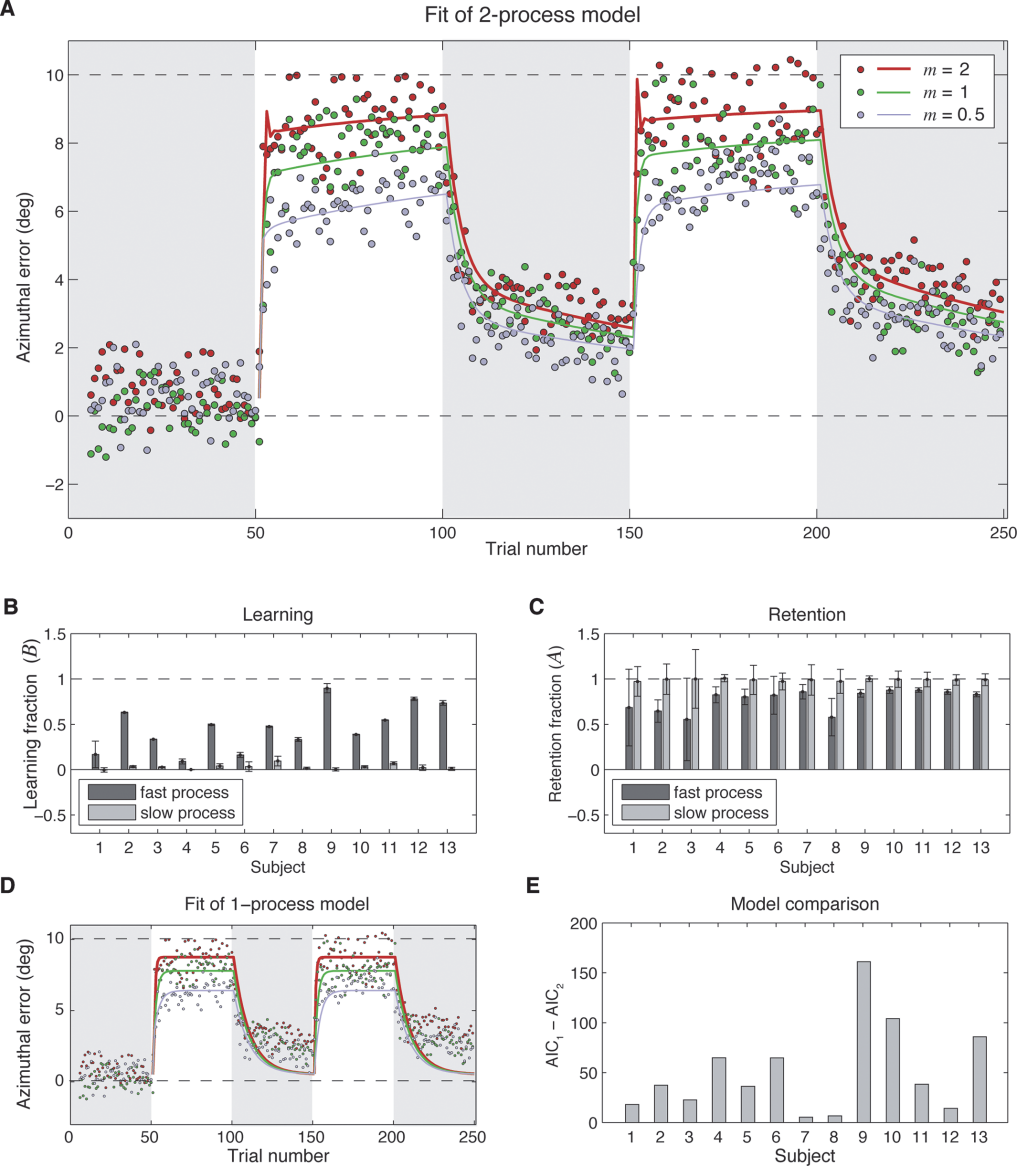
Results. **(A)** Mean azimuthal errors as a function of trial number for the three magnification conditions (*m* = 2, *m* = 1, *m* = 0.5), together with a model (eq. 3) with the mean fit parameters of the 13 subjects. Gray background indicates the blocks without visual feedback. **(B)** Model comparison. Same data as in A, with a one-process model (*θ*_*i*+1_ = A*θ_*i*_ + B * e_*i*_) with mean fit parameters. **(C)** Comparison of the Akaike information Criterion for the two-process model (AIC_2_) and one-process model (AIC_1_). For all subjects, the AIC was lower for the two-process model, indicating that it was a better description of the data. **(D)** Fit learning fractions (*B*_1_, dark grey bars; *B*_2_, light grey bars) with 95% confidence intervals. **(E)** Fit retention fractions (*A*_1_, dark grey bars; *A*_2_, light grey bars) with 95% confidence intervals.

We studied adaptation by measuring the time course of azimuthal errors under three levels of error magnification. Adaptation was incomplete: it always leveled off before reaching 10° (Fig. 2.A). In the magnification conditions in which *m* was 2 or 1, some subjects spontaneously reported that the feedback was perturbed. When *m* was 0.5, none of the subjects commented on the feedback being perturbed.

To start with, we examined how the level of adaptation (the mean azimuthal error in the last ten trials of the feedback blocks) depended on the magnification. As predicted, there was a main effect of the magnification on the level of adaptation (*F*(2,11) = 11.43, *p* < 0.001). When errors were not magnified (*m* = 1), adaptation leveled off at 79% correction for the perturbation. Magnifying errors by a factor of 2, we raised this level to 90%. Scaling errors down with a magnification factor of 0.5, reduced the level to about 67%. Thus, the larger the magnification factor, the more complete the adaptation (Fig. 2.A). There was no main effect of the repetition (*F*(1,12) = 0.01, *p* = 0.92) and also no interaction between magnification and repetition (*F*(2,11) = 1.2, *p* = 0.32).

To examine whether the magnification of the visible errors influenced the learning and retention fractions, we first fit the model to the azimuthal errors in the feedback and post-feedback blocks of each of the three magnification conditions separately, and compared the learning and retention fractions across magnification conditions. We used a Kruskal-Wallis non-parametric test of equality of medians because there were outliers in the estimated model parameters for two subjects, due to unreliable model fits. In accordance with our assumption that the magnification of visible errors would not affect the learning fraction, none of the learning or retention fractions depended on the magnification factor (with the test results for the parameters *A*_*1*_, *B*_*1*_, *A*_*2*_ and *B*_*2*_ being respectively: *X* ^2^ (2,36) = 0.72, *p* = 0.7; *X* ^2^ (2,36) = 1.51, *p* = 0.47; *X* ^2^ (2,36) = 1.61, *p* = 0.45; and *X* ^2^ (2,36) = 0.36; *p* = 0.84). Note that in our model, for a constant learning fraction, magnifying the error will result in faster adaptation.

As there was no influence of the magnification factor on the learning and retention fractions, we further rely on the model fit to the individual subjects’ azimuthal errors in the feedback and post-feedback blocks of all three magnification conditions with a single set of four parameters (Fig. 2.A). These model fits provided estimates of how learning (*B*_1_, *B*_2_; Fig. 2.B) and retention (*A*_1_, *A*_2_; Fig. 2.C) contributed to the adaptation. As can be seen in Fig. 2.A, the model provided a good description of how the average errors depended on the (magnified) visual feedback. The model predicted that the larger magnification factor would make subjects overcompensate for their errors in the first few trials of a feedback block, resulting in corrections in the opposite direction in the next trial (see black curve in Fig. 2.A). The predicted size of this effect is however small compared to the response variability, which is probably why this is not visible in the data.

To check whether the subjects’ adaptation was best described by a two-process model rather than by a simpler single-process model, we also fit a one-process version of the Smith Model, with a single learning and forgetting fraction, to the subjects azimuthal errors. As was done for the two-process model, we first checked whether the model parameters depended on the magnification factor by fitting the model to the data of the three magnification conditions. Comparison of the learning and retention fractions showed that there were no differences between the fit parameters in the different magnification conditions.

Fig. 2.D shows the model fit to data of all three magnification conditions. As can be seen, this model did not capture the fact that in blocks with feedback errors do not return to baseline. To statistically confirm that the two-process model provided the better fit, we compared the models with the Akaike Information Criterion, that corrects for the number of parameters. For all subjects, the Akaike Information Criterion was lower for the two-process model, indicating that the two-process model better explained the data (Fig. 2.E).

Do both processes learn and forget? If there is both learning and forgetting, *B* should be greater than 0 whereas *A* should be smaller than 1 (forgetting is incomplete retention). To test whether this is so, we entered the parameter estimates in separate one-tailed t-tests. For learning (Fig. 2.B), we found that *B*_1_ was on average 0.46 (significantly larger than 0; *t*(12) = 6.67, *p* < 0.001), and *B*_2_ was on average 0.03 (also significantly larger than 0; *t*(12) = 3.69, *p* = 0.003). Thus, there was significant learning in both processes. For retention (Fig. 2.C), we found that *A*_1_ was on average 0.77 (significantly smaller than 1; *t*(12) = -7.10, *p* < 0.001) and *A*_2_ was on average 0.99 (also significantly smaller than 1; *t*(12) = -3.14, *p* = 0.009). Thus, there was also significant forgetting in both processes. The fast process corrected for 46% of the error on each trial, but forgot 23% of what it had learnt. The slow process corrected for 3% of the error on each trial and forgot 1% of what it had learnt.

**Fig 3:**
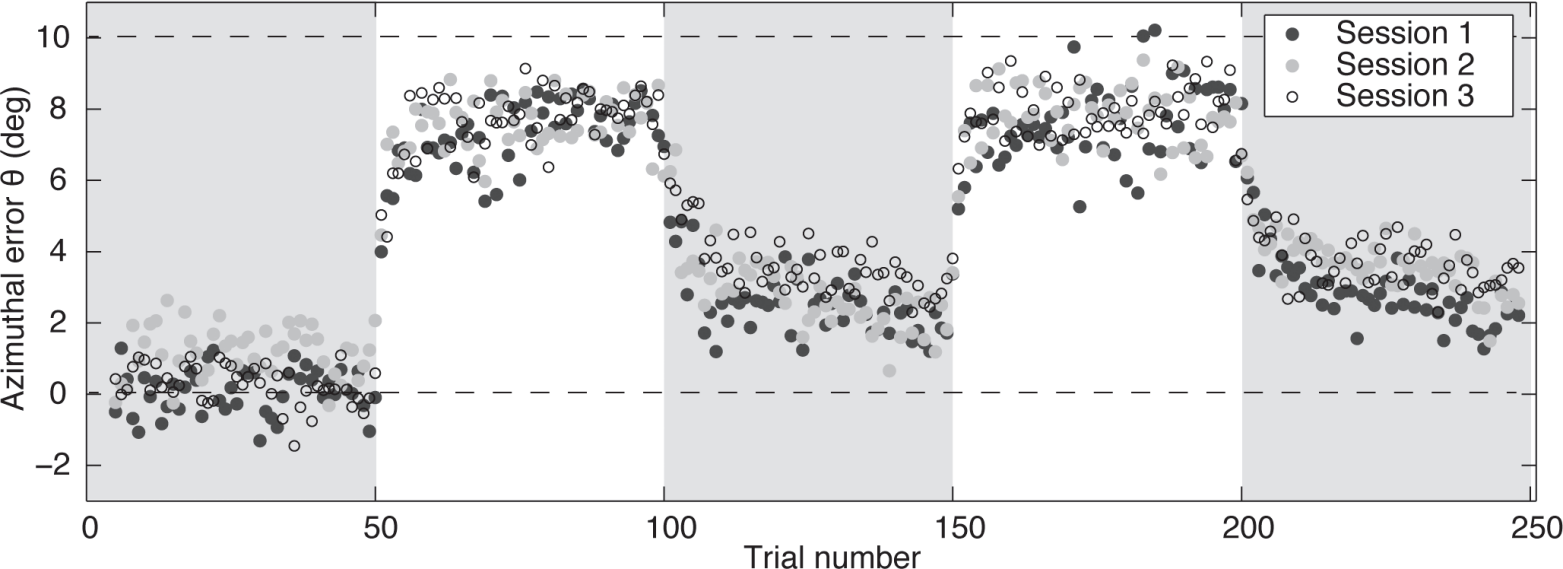
Order effects. Azimuthal errors as a function of trial number when averaged by session rather than by magnification condition. The session number did not influence the level of adaptation in feedback blocks.

The exact values of the parameters need to be interpreted with caution. We used a within-subjects design, which allowed us to test the prediction that the balance between learning and forgetting could explain the different adaptation in the three magnification conditions. However, using a within-subjects design introduced the possibility of transfer of learning between conditions. We accounted for a change in the baseline bias by determining the initial bias in the pre-feedback block for each session. However, there may have been other transfer effects, for instance on the rate at which the mapping is learnt (savings). To check whether there was transfer of adaptation between sessions, we re-grouped the data by the order in which sessions had been performed (Fig. 3) and re-performed the repeated measures analysis on the level of adaptation during the last 10 trials of the feedback blocks, this time with the factor Session Number rather than Magnification. This repeated measures analysis showed that there was no significant influence of the Session Number on the level of adaptation (*F*(2,11), = 0.18, *p* = 0.83).

## Discussion

In this study, we examined why adaptation is generally incomplete. In doing so, we focused on the idea that the incompleteness of adaptation is caused by forgetting: the learnt adaptation is already partially forgotten by the time the next movement is made [5,14,19]. We tested this idea by scaling the shown errors with three different magnification factors (2, 1, 0.5), perturbing the balance between learning and forgetting.

As predicted, we found that adaptation to a -10° rotational bias in the visual feedback was more complete when errors were scaled with a larger magnification factor. This result provides the first experimental support for the idea that incompleteness of adaptation is caused by adaptation involving forgetting as well learning. In contrast to reports in the literature that the learning fraction may depend on the size of errors [30–32], we did not find that the learning fraction depended on the magnification factor. An alternative explanation of how the level of adaptation in blocks with feedback depended on the scaling of errors is that subjects maintained a constant visual error. However, it is unclear why they would do so. Moreover, such an explanation requires several quite arbitrary parameters to account for forgetting in blocks without feedback. In the following paragraphs we therefore proceed to discuss in more detail how the incompleteness of adaptation can be explained by the forgetting and why there would be such forgetting.

To study in detail how the course of errors in blocks with and without feedback can be described by a combination of learning and forgetting, we fit the Smith et al. (2006) model of adaptation that has two processes that learn and forget at different rates to the movement errors. We chose to use this model because it provides a simple but adequate description of how learning and forgetting contribute to adaptation. The model has been shown to be limited in explaining adaptation in more complex situations than the one we tested. For instance when subjects receive both veridical and perturbed feedback [26] or adapt to a set of different perturbations [27–28] or when episodes in which subjects see their errors are alternated with episodes in which subjects only receive binary information about movement success [29]. We did not observe any systematic deviations from the model’s predictions.

The model fit shows that the same learning and retention fractions can explain the different levels of adaptation found for the three magnification factors throughout feedback and post-feedback blocks. There was significant learning in both processes. The retention fraction of both processes was smaller than 1, indicating that there was significant forgetting in both processes. However, the retention fraction of the first process was much lower than that of the second process, indicating that fast learning was associated with fast forgetting whereas slow learning was associated with good retention.

Our results confirm the idea that incomplete adaptation during blocks with feedback can be explained by the same forgetting that describes the return to baseline in blocks without feedback. This finding is important because it provides insight in why adaptation is incomplete. The phenomenon that has often been treated as a mere imperfection of human adaptive abilities but may in fact serve a function in our interaction with the environment. An important question is therefore why the nervous system counteracts fast learning with fast forgetting whilst retaining what has been learnt slowly. If adaptation were handled by a single volatile process that learns and forgets rapidly, the forgetting would prevent us from ever learning anything permanently. Yet, we seem able to adjust permanently to the changes that belong to the transformation to the adult body. Ability to deal with such gradual but lasting changes can be attributed to the slow process, which learns slowly but retains what it has learnt well. The interplay between the fast and slow process allows us to rapidly adjust to sudden changes whilst remaining able to return to a long-proven baseline [30]. The forgetting can thus be considered a sign of conservatism rather than oblivion.

Being conservative in adapting the nervous system is not unwise: many changes are transient, for instance when they are due to external perturbations [31,32] or fatigue. The fast process is suited to adapt to such changes: it learns quickly but forgets corrections rapidly when perturbations pass. Lasting changes in the sensory mappings, such as due to bodily growth or degeneration, typically occur gradually and can be learnt by the slow process, which retains its information well.

Finally, the recent literature has questioned the idea that the fast and slow processes learn in a similar way from performance errors [21]. Introducing an episode of binary ‘reinforcement’ feedback about movement success has been found to influence the adaptation. That is, introducing an episode of reinforcement feedback once subjects had achieved their maximal level of adaptation caused errors in post-feedback blocks to level off at a higher level of adaptation than when subjects saw their errors during this period [22]. As the point at which errors level off in post-feedback blocks coincides with what has been learnt by the slow process, this suggests that the slow process may be a form of *reinforcement learning* [22]. Does this mean that reinforcement learning is the mechanism that keeps unbiased performance within reach? That remains unclear. Reinforcement learning is held to rely on storing successful movements rather than adjusting a forward model, and all studies that attribute persistent components of adaptation to reinforcement learning have used paradigms in which movements were repeated across trials [21,22]. In our study subjects made different movements on each trial and the only constant was the 10° visual perturbation they had to adapt to. Reinforcement learning could thus have only contributed to the adaptation if, rather than a specific movement, a correction to the movement planning was reinforced.

Understanding how learning and forgetting contribute to adaptation is important for the development of rehabilitation paradigms. Because persistent adaptation relies on slow learning, rehabilitation takes long and is hard work. Patients could thus benefit greatly from methods that facilitate the adaptation. Our results show that learning can be boosted by magnifying errors, an idea that has also been explored by others [33]. However, we saw no evidence of a lasting benefit of the boosted learning. Moreover subjects spontaneously reported disbelief in the large errors they saw, displaying annoyance with the experiment. Perhaps patients benefit more from methods that reduce the forgetting. Interestingly, the recent literature suggests that the learning and forgetting may be conceived as switching between contextual states rather than the acquisition and decay of single state [27,28, 34]. This implies that the forgetting during blocks with feedback is caused by uncertainty on the contextual state that should be applied and that the forgetting is facilitated by contextual cues that a different context applies [35]. Virtual rehabilitation paradigms may therefore benefit from minimizing differences between the training and real-life context.

To conclude, we found experimental evidence for the idea that adaptation is incomplete because part of what has been learnt is forgotten quickly. Does the forgetting mean that when injury induces large biases in our movements we can never fully recover from this? No, in addition to fast adaptive processes that learn and forget quickly, there are also slow processes that learn slowly but retain what they have learnt well. Thus, we can recover fully but very slowly. Therefore understanding the causes of forgetting may enable the development of rehabilitation paradigms that allow patients to rehabilitate faster.
